# Ethanol Extract of *Evodia rutaecarpa* Attenuates Cell Growth through Caspase-Dependent Apoptosis in Benign Prostatic Hyperplasia-1 Cells

**DOI:** 10.3390/nu10040523

**Published:** 2018-04-22

**Authors:** Eunsook Park, Mee-Young Lee, Chang-Seob Seo, Ji-Hye Jang, Yong-ung Kim, Hyeun-Kyoo Shin

**Affiliations:** 1 Korea Institute of Oriental Medicine, 1672 Yuseong-daero, Yuseong-gu, Daejeon 34054, Korea; eunsook@kiom.re.kr (E.P.); cozy11@kiom.re.kr (M.-Y.L.); csseo0914@kiom.re.kr (C.-S.S.); jihye1684@kiom.re.kr (J.-H.J.); 2 Department of Pharmaceutical Engineering, College of Biomedical Science, Daegu Haany University, 1 Hanuidae-ro, Gyeongsan-si, Gyeongsangbuk-do 38610, Korea

**Keywords:** 5α-reductase activity, apoptosis, Benign prostatic hyperplasia, cell growth, *Evodia rutaecarpa* Bentham

## Abstract

The dried fruits of *Evodia rutaecarpa* Bentham have been used widely as a herbal medicine for the treatment of inflammatory disorders and abdominal pain. Benign prostatic hyperplasia (BPH) is a nonmalignant disease characterized by overgrowth of prostates. Despite the pharmacological efficacy of the fruits of *E. rutaecarpa* against various diseases, their effects against BPH have not been reported. Here, we investigated the inhibitory activity of a 70% ethanol extract of *E. rutaecarpa* (EEER) against BPH, and its underlying mechanisms regarding cell growth of BPH using BPH-1 cells. An in vitro 5α-reductase activity assay showed that EEER exhibited inhibitory activity against 5α-reductase. In BPH-1 cells, EEER treatment inhibited cell viability and reduced the expression of the proliferating cell nuclear antigen proliferating cell nuclear antigen (PCNA), cyclin D1, and phosphor-ERK1/2 proteins. Moreover, EEER also induced apoptosis, with chromatin condensation, apoptotic bodies, and internucleosomal DNA fragmentation. Regarding its underlying mechanisms, EEER exacerbated the activation of caspase-8 and caspase-3 in a concentration-dependent manner and eventually caused the cleavage of PARP. Taken together, these data demonstrated that EEER had a potent 5α-reductase inhibitory activity and that EEER treatment in BPH-1 cells inhibited cell viability via caspase-8- and caspase-3-dependent apoptosis. Therefore, EEER may be a potential phytotherapeutic agent for the treatment of BPH.

## 1. Introduction

The dried fruits of *Evodia rutaecarpa* Bentham (Rutaceae), Evodiae fructus, are a traditional herbal medicine commonly used in Asia as an analgesic, antiemetic, hemostatic, antihypertensive, and uterotonic agent [1]. Recently, several studies have demonstrated the pharmacological properties of *E. rutaecarpa* in several disorders. *E. rutaecarpa* extract had an inhibitory effect on both intestinal transit and diarrhea in mice [2,3]. An ethanol extract of *E. rutaecarpa* exhibited antioxidant and anti-inflammatory activities in an endotoxemic rat model and in microglial cells [4,5]. An *E. rutaecarpa* extract was also effective in alleviating alcohol-induced hangover symptoms in a mouse model [6]. Furthermore, an ethanol extract of *E. rutaecarpa* had antitumor activity in cervical cancer cells [7]. Based on the multiple therapeutic activities of *E. rutaecarpa*, including anti-inflammatory, antioxidative, and antiproliferative effects, a potential therapeutic action of *E. rutaecarpa* against a variety of diseases is expected.

Benign prostatic hyperplasia (BPH), which is one of the most common diseases in men aged >50 years, is a nonmalignant disease that is clinically characterized by an enlargement of the prostate gland [8]. BPH results from the overproliferation of the cellular components of the transition zone of the prostate surrounding the proximal urethra and is eventually accompanied by lower urinary tract symptoms, including disorder of urinary storage and voiding and symptoms after urination [9]. Although the cause of BPH has not been defined, it is well known that the incidence of BPH is associated with higher levels of dihydroxytestosterone (DHT), a hormone that is related to cellular proliferation in the prostate, but not of testosterone [10,11]. The 5α-reductase inhibitors finasteride and dutasteride, which block the conversion of testosterone into DHT, are used effectively as pharmacological therapies to improve BPH regarding the reduction of prostate volume [12,13]. However, many patients treated with these pharmacological therapies suffer simultaneous adverse effects, such as orthostatic hypotension, hair loss, and sexual dysfunction [14,15]. Recently, therefore, many researchers have been attempting the development of phytotherapeutic agents composed of herbal medicines, which have structural diversity and less toxicity compared with pharmacological agents [16].

Regarding the development of a phytotherapeutic treatment for BPH, our group was interested in the antiproliferative property of *E. rutaecarpa*. In the present study, we investigated the inhibitory effect of an ethanol extract of *E. rutaecarpa* (EEER) against cell growth in BPH using human BPH epithelial (BPH-1) cells and identified the mechanisms underlying the effect of EEER in BPH-1 cells.

## 2. Material and Methods

### 2.1. Plants, Chemicals, and Reagents

The dried fruits of *Evodia rutaecarpa* Bentham (Rutaceae) were purchased from the herbal market Kwangmyungdang Medicinal Herbs (Ulsan, Korea) in June 2016 and identified by Dr. Goya Choi, K-herb Research Center, Korea Institute of Oriental Medicine (KIOM; Daejeon, Korea). A voucher specimen (2016-EBM-104-1) has been deposited at the K-herb Research Center, KIOM.

**Figure 1:**
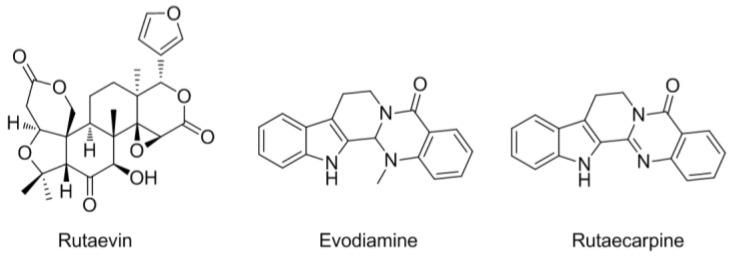
Chemical structure of the three bioactive marker compounds of *E. rutaecarpa*.

For chemical profiling, three reference standard compounds, rutaevin (PubChem CID: 441805; purity, 95.7%), evodiamine (PubChem CID: 442088; purity ≥99.0%), and rutaecarpine (PubChem CID: 65752; purity, ≥99.0%) were purchased from Chengdu Biopurify Phytochemicals Ltd. (Chengdu, China), Merck KGaA (Darmstadt, Germany), and Shanghai Sunny Biotech Co., Ltd. (Shanghai, China), respectively. The chemical structures of the three reference compounds are shown in Figure 1. High-performance liquid chromatography (HPLC)-grade solvents, methanol, acetonitrile, and water were obtained from J.T. Baker (Phillipsburg, NJ, USA). For the in vitro assay, the anti-PCNA and anti-cyclin D1 antibodies were purchased from Abcam (Cambridge, UK). The anti-phospho-ERK1/2 (Thr202/Tyr204), anti-ERK1/2, and anti-poly(ADP-ribose) polymerase (PARP) antibodies were from Cell Signaling (Danvers, MA, USA). The anti-β-actin antibody was purchased from Santa Cruz Biotechnology (Santa Cruz, CA, USA).

### 2.2. Preparation of a 70% Ethanol Extract of E. rutaecarpa (EEER)

The fruits of *E. rutaecarpa* (20.0 kg) were extracted with 70% ethanol (200 L) for 3 h at 80 °C using an electric extractor (KSP-240L; Kyungseo Machine Co., Incheon, Korea). To remove the organic solvent, the extract solution was concentrated using an EV-1020 Digital Rotary evaporator (SciLab Korea Co. Ltd., Seoul, Korea) under vacuum and freeze dried (Genesis 25LL, Virtis Co., Gardiner, NY, USA), to give a powdered sample. The amount of lyophilized 70% ethanol extract was 5.36 kg (26.8%). A lyophilized sample was dissolved in dimethyl sulfoxide (DMSO) for the in vitro assay.

### 2.3. HPLC Analysis of the Three Marker Components of E. rutaecarpa

An HPLC analysis for the quantitative assessment of the three marker compounds of *E. rutaecarpa* was conducted on a Shimadzu Prominence LC-20A series HPLC (Shimadzu, Kyoto, Japan), consisting of two delivery systems (LC-20AT), an online degasser (DGU-20A_3_), a column oven (CTO-20A), an auto-sampler (SIL-20AC), and a photo-diode array (PDA) detector. All data were acquired and processed using the LabSolution software (Version 1.24 SP1, Kyoto, Japan). The three bioactive marker compounds of *E. rutaecarpa* were separated on a Phenomenex Gemini C_18_ column (250 × 4.6 mm, 5 μm, Torrance, CA, USA) at 40 °C. The mobile phase consisted of distilled water and acetonitrile and was flowed under isocratic elution of 40% acetonitrile for 50 min. The flow rate was 1.0 mL/min and the injection volume was 10 μL. For quantitative determination of the triterpenoid (rutaevin) and the two alkaloids (evodiamine and rutaecarpine) in *E. rutaecarpa*, 200 mg of a lyophilized *E. rutaecarpa* powdered sample was dissolved in 20 mL of 70% methanol and sonicated for 60 min at room temperature using a Branson ultrasonicator (8510E-DTH; Danbury, CT, USA). Subsequently, the extract solution was filtered through a 0.2 μm syringe filter (Pall Life Sciences, Ann Arbor, MI, USA) before HPLC analysis.

### 2.4. 5α-Reductase Activity

In vitro 5α-reductase inhibitory activity was measured as described previously [17]. Briefly, the suspension of testosterone 5α-reductase was prepared from the homogenate of the ventral prostates of male Sprague Dawley rats. The reaction mixture including the sample, the suspension of testosterone 5α-reductase, NADPH, and (1,2,6,7-^3^H)-testosterone was incubated for 1 h at 37 °C, and ^3^H radioactivity was counted using a liquid scintillation counter (Beckman Coulter, Brea, CA, USA). Each enzymatic reaction was carried out in duplicate.

### 2.5. Cell Culture

The human BPH epithelial cell line BPH-1 was obtained from Creative Bioarray (Shirley, NY, USA). BPH-1 cells were maintained in RPMI 1640 medium supplemented with 20% FBS (Invitrogen, Carlsbad, CA, USA) and 1% antibiotics, and incubated in a humidified 5% CO_2_ atmosphere at 37 °C [18].

### 2.6. Cell Growth Assay

To examine cell growth, a Cell Counting Kit (CCK)-8 assay (Dojindo, Tokyo, Japan) was performed as described previously [18]. Briefly, BPH-1 cells, which were seeded at 3.5 × 10^3^ cells/well and 2.5 × 10^3^ cells/well on a 96-well plate, were treated with a concentration series of EEER and incubated for an additional 24 or 48 h.

### 2.7. Western Blot Analysis

Whole-cell lysates from BPH-1 cells were prepared in RIPA lysis buffer (Sigma-Aldrich, St. Louis, MO, USA). Equal amounts of protein were electrophoresed on a mini-PROTEAN TGX precast gel (Bio-Rad Laboratories Inc., Hercules, CA, USA) and transferred to a PVDF membrane (Millipore, Billerica, MA, USA). After incubation with antibodies, proteins were visualized using the SuperSignal West Femto Maximum Sensitivity Substrate (Thermo Fisher, Waltham, MA, USA) and a ChemiDOC XRS^+^ detection system (Bio-Rad, Hercules, CA, USA).

### 2.8. DNA Fragmentation Assay

Internucleosomal DNA fragmentation was assessed using the Apoptotic DNA Ladder Detection kit (Abcam), according to the manufacturer’s instructions. Briefly, BPH-1 cells (5 × 10^5^ cells) were harvested and genomic DNA was precipitated with isopropanol. The resuspended DNA was separated on a 1.7% agarose gel. The DNA fragmentation was visualized by staining with Loading STAR (DyneBio, Seongnam, Korea).

### 2.9. Nuclear Staining with DAPI

After the treatment with EEER, BPH-1 cells were harvested and fixed with 4% paraformaldehyde for 10 min. The fixed cells were incubated with 1 μM of 6-diamidino-2-phenylindole (DAPI; Sigma-Aldrich) for 5 min. The stained cell nuclei were analyzed on a Nikon ECLIPSE Ts2 fluorescence microscope (Nikon Inc., Melville, NY, USA).

### 2.10. Determination of Caspase Activity

Caspase-3, caspase-8, and caspase-9 colorimetric assay kits (BioVision, Mountain View, CA, USA) were used to detect the activity of the respective caspases. Briefly, BPH-1 cells (3 × 10^6^ cells) were collected and lysed in lysis buffer. Cytosolic extracts (200 μg) were incubated with reaction buffer, dithiothreitol, and the respective substrate at 37 °C. Caspase activity was determined through the measurement of absorbance at 405 nm using a microplate reader (Bio-Rad).

### 2.11. Statistical Analysis

Values were presented as the mean ± standard error of the mean (SEM). Quantitative data were calculated using a one-way analysis of variance (ANOVA) with Dunnett’s multiple comparisons. *p* < 0.05 was considered statistically significant.

## 3. Results

### 3.1. HPLC Analysis of the Three Marker Components of EEER

**Figure 2:**
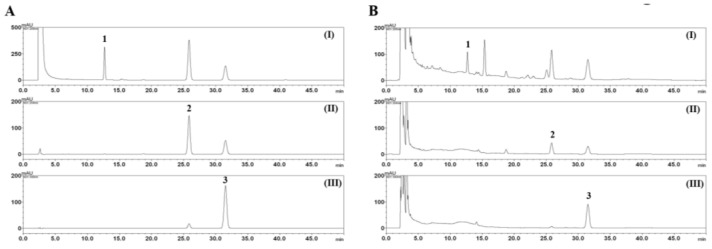
Quantitative analysis of the three marker compounds of ethanol extract of *E. rutaecarpa* (EEER). HPLC chromatograms of the standard mixture (**A**) and EEER (**B**) at a UV wavelength of 205 nm (I), 254 nm (II), and 340 nm (III). Rutaevin (**1**), evodiamine (**2**), and rutaecarpine (**3**).

The established HPLC–PDA analytical method was applied to the quantitative analysis of the triterpenoid (rutaevin) and two alkaloids (evodiamine and rutaecarpine) present in EEER. Consequently, three marker compounds, rutaevin, evodiamine, and rutaecarpine, were eluted within 35 min and detected at 12.67, 25.84, and 31.51 min, respectively. HPLC–PDA chromatograms of the standard mixture and EEER are shown in Figure 2B. The calibration curves of rutaevin, evodiamine, and rutaecarpine were *y* = 7664.43*x* − 1846.44, *y* = 17,195.72*x* − 2688.32, and *y* = 119,380.78*x* − 31,768.37, respectively, with a correlation coefficient ≥0.9999. Based on these results, the amount of the three bioactive marker compounds (rutaevin, evodiamine, and rutaecarpine) of EEER was calculated as 13.25, 6.12, and 2.34 mg/g, respectively.

### 3.2. EEER Suppresses 5α-Reductase Activity

To evaluate the potential activity of EEER against BPH, we initially examined the regulation of 5α-reductase activity by EEER. As shown in Table 1, 37.2 ng/mL of finasteride as a positive control yielded high inhibition of 5α-reductase activity (89.3 ± 15.4%). Remarkably, 25 μg/mL of EEER exhibited an outstanding efficacy (80.2 ± 11.4%) in the inhibition of 5α-reductase, similar to that of finasteride.

### 3.3. EEER Inhibits the Growth of BPH-1 Cells

**Figure 3:**
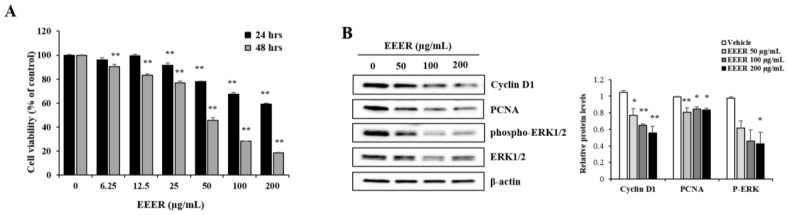
Effect of EEER on the growth of BPH-1 cells. (**A**) Cells were treated with vehicle or EEER for 24 and 48 h at the indicated concentration and the relative cell viability was assessed using the CCK-8 assay. DMSO was used as the vehicle. (**B**) The assessment of protein expression levels was performed via western blot analysis (**left**). The relative cyclin D1 and PCNA levels and phospho-ERK1/2 levels were normalized to that of β-actin and ERK1/2 levels, respectively. The densitometric analysis was performed using the Image Lab software (**right**). The data are the mean ± SEM of three independent experiments. * *p* < 0.05 and ** *p* < 0.01 compared with the vehicle control.

To assess whether EEER affects the growth of BPH cells, the viability of BPH-1 cells was determined via CCK-8 assay. EEER treatment for 24 h in BPH-1 cells resulted in the reduction of cell viability in a concentration-dependent manner, and this result was exacerbated by the 48-h treatment (Figure 3A). Moreover, the reduced cell viability observed after treatment with EEER was correlated with changes in the expression of proliferation-related proteins. Western blot analysis showed that EEER strictly suppressed the levels of the cyclin D1, PCNA, and phosphorylated ERK1/2 proteins (Figure 3B).

### 3.4. EEER Induces Apoptosis in BPH-1 Cells

**Figure 4:**
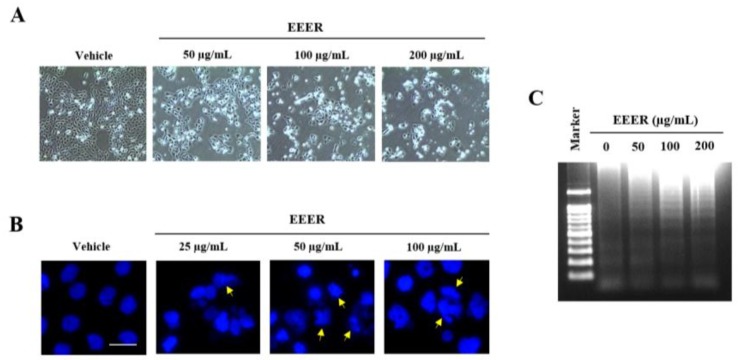
Effect of EEER on apoptosis in BPH-1 cells. Cells were treated with vehicle or EEER for 24 h at the indicated concentrations. (**A**) Representative images of cell confluence and morphology observed under a phase-contrast microscope (magnification 100×). (**B**) Representative images of DAPI-stained cells observed under a fluorescence microscope (scale bar, 20 μm). The arrows indicate signs of apoptotic morphology, including chromatin condensation and apoptotic bodies. (**C**) Fragmentation of genomic DNA as analyzed by agarose gel electrophoresis. Images are representative of three independent experiments.

As assessed using phase-contrast microscopy, the decrease in cell confluence observed after EEER treatment at 50 μg/mL in BPH-1 cells was accompanied by morphological changes, such as cellular shrinkage and rounding of the cell shape; EEER treatment at 100 and 200 μg/mL led to cell death (Figure 4A). To determine whether the cell death induced by EEER was caused by apoptosis, apoptotic characteristics were further examined in detail. In the nuclei of BPH-1 cells, treatment with EEER, but not vehicle, resulted in chromatin condensation and formation of apoptotic bodies (Figure 4B). In addition, DNA fragmentation was observed in EEER-treated BPH-1 cells in a concentration-dependent manner (Figure 4C).

### 3.5. EEER-Induced Apoptosis Involves Caspase-8 and Caspase-3 Activation in BPH-1 Cells

**Figure 5:**
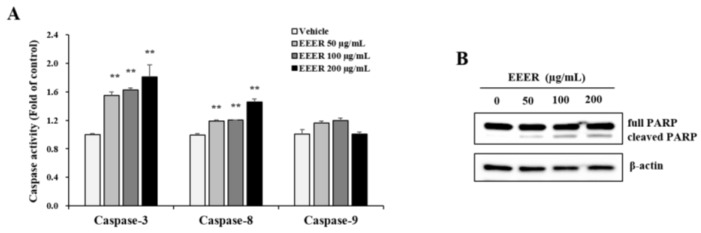
Effect of EEER on caspase activation in BPH-1 cells. (**A**) Caspase-3, caspase-8, and caspase-9 activities. Cytosolic extracts (200 μg) from cells treated with vehicle or EEER for 24 h were subjected to caspase-3, caspase-8, and caspase-9 colorimetric assays; (**B**) The protein levels of total PARP and cleaved PARP were assessed by western blot analysis. Data are representative of three independent experiments performed in triplicate. ** *p* < 0.01 compared with the vehicle control.

To address further the mechanisms by which EEER induces apoptosis, we examined the association of caspase activation with EEER-induced apoptosis. Caspase-3 activity was remarkably increased after EEER treatment (Figure 5A). Among the upstream mediators of caspase-3 activation, caspase-8 was consistently activated by EEER treatment, whereas caspase-9 was rarely affected by EEER administration (Figure 5B). The PARP enzyme, which is one of the substrates that are cleaved by caspase-3, is a key mediator of apoptosis [19]. Western blot analysis revealed that the PARP protein was cleaved by EEER treatment at 50 μg/mL, and its cleavage was gradually accelerated by increasing the concentration of EEER (Figure 5B).

## 4. Discussion

The enzyme 5α-Reductase, which converts testosterone to DHT, comprises type 1, 2, and type 3 isoenzymes and plays an important role in BPH progression [12]. In this study, we first assessed whether EEER affects 5α-reductase activity. 25 μg/mL of EEER showed 80% inhibition of 5α-reductase activity which was appeared by 37 ng/mL of finasteride, an inhibitor of this enzyme (Table 1). Unlike the 5α-reductase type 3 isoenzyme, which is mainly expressed in hormone-refractory prostates, the type 1 and type 2 isoenzymes are expressed at low and high levels in normal prostates, respectively [20]. The suspension of testosterone 5α-reductase used in this study was obtained from the ventral prostates of male rats, which indicates that the inhibitory activity of EEER on 5α-reductase might involve type 1 or type 2 isoenzymes, at least. Based on the results presented above, we investigated in depth the effect of EEER on cell growth in BPH, as well as the underlying mechanisms.

The major pathological feature of BPH, i.e., the abnormal growth of prostate cells, is caused by an imbalance between proliferation and cell death [21]. Cyclin D1 and PCNA, which are major proteins in the control of cell-cycle progression, are considered to be reliable indicators of the proliferation rate [22,23]. In addition, ERK1/2 signaling plays a role in various stimulus-induced cellular responses, including proliferation, and its phosphorylation mediates the increase of PCNA and cyclin D1 expression [24]. Consistently, EEER administration to BPH-1 cells inhibited cell viability, which was accompanied by reduced levels of the phosphorylated ERK1/2 protein, as well as PCNA and cyclin D1 (Figure 3B), indicating that EEER repressed the growth of BPH-1 cells.

Cell death acts as an essential factor in many pathologies and biological processes. Two main types of cell death exist, apoptosis and necrosis [25]. Apoptosis is a programmed cell death that occurs under normal conditions for tissue homeostasis, embryogenesis, and immunity and is activated through the apoptotic signal transduction pathway, including proteolytic processes of caspase proteins. Dysregulation of apoptosis leads to many diseases, including autoimmune disorders, neurodegenerative diseases, and cancer [26]. In contrast, necrosis is an accidental cell death that is caused by external factors, including infection and exhaustion of oxygen or nutriment, and results in extensive tissue damage and inflammatory response [27].

Cells undergoing apoptosis or necrosis are distinguished by different characteristics regarding morphological and biochemical changes [28]. Morphologically, apoptotic cells exhibit plasma membrane blebbing, chromatin condensation in nuclei, and apoptotic bodies, whereas necrotic cells present cellular lysis and swelling. In the present study, EEER-treated BPH-1 cells showed cell death with morphological features that included chromatin condensation and apoptotic bodies, as well as a round shape (Figure 4A,B). Regarding biochemical features, apoptosis, but not necrosis, involves the release of cytochrome C and the activation of the caspase cascade. Although both types of cell death induce DNA fragmentation, apoptosis is accompanied by regular-length fragments, whereas necrosis results in fragments with a random length and diffuse fragments. Therefore, these different features have been described as hallmarks of apoptosis and necrosis [29]. EEER-treated BPH-1 cells also exhibited specific biochemical features, such as a ladder pattern of DNA fragmentation and caspase activation (Figure 4C and Figure 5A), indicating that EEER-induced cell death involves apoptosis, and not necrosis.

Apoptosis involves the activation of caspases and PARP, a processing of proteolytic cleavages, via extrinsic or intrinsic pathway [27,30,31]. Caspases are divided into two subtypes, initiator and executioner caspases: initiator caspases (caspase-2, caspase-8, and caspase-9) are first activated, then their cleavage leads to the activation of executioner caspases (caspase-3, caspase-6, and caspase-7). PARP is cleaved by active executioner caspases, and cleaved PARP induces the acceleration of the apoptotic process, including DNA fragmentation [32]. The extrinsic pathway, which is initiated by the activation of the extracellular death receptor, induces caspase-8 activation via the assembly of the death-inducing signaling complex. Activated caspase-8 then instigates apoptosis through direct caspase-3 activation or the Bid cleavage-mediated mitochondrial pathway. Conversely, the intrinsic pathway involves the release of cytochrome c via mitochondrial outer-membrane permeabilization, and cytosolic cytochrome c assembles the apoptosome complex with caspase-9 activation. Activated caspase-9 directly induces caspase-3 activation, leading to apoptosis. Regarding caspase activation, EEER administration to BPH-1 cells induced the activation of both caspase-3 and caspase-8, but not caspase-9, as well as PARP cleavage (Figure 5), indicating that EEER-induced apoptosis is associated with caspase-8 activation through the extrinsic pathway.

There are three major bioactive compounds in the dried fruits of *Evodia rutaecarpa* Bentham [33]. Rutaevin, evodiamine, and rutaecarpine were determined quantitatively in the EEER used in this study (Figure 1 and Figure 2). Rutaecarpine exhibits pharmacological properties, including a cardiovascular effect, antiplatelet activity, and anti-inflammatory activity [34]. Evodiamine has a robust anticancer bioactivity via the inhibition of cell proliferation or the induction of apoptosis [35,36]. Considering the molecular functions of these compounds, we suggest that the EEER efficacy in BPH-1 cells may be associated with the evodiamine compound. Several studies have demonstrated that evodiamine-induced apoptosis is associated with caspase-9 activation through mitochondrial dysfunction [37,38]. In prostate cancer cells, however, evodiamine caused an elevation in the activities of caspase-3 and caspase-9, but not caspase-8, in PC3 and LNCaP cells; and of caspase-3, caspase-9, and caspase-8 in DU145 cells [39,40]. In thyroid, ovarian, and colon cancer cells, evodiamine led to the activation of caspase-3, caspase-8, and caspase-9 [41,42,43]. Therefore, evodiamine-induced apoptosis via the extrinsic or intrinsic pathway might be flexible, depending on cell microenvironment. Furthermore, we showed that the administration of EEER to BPH-1 cells activated caspase-8 and caspase-3, but not caspase-9. The studies mentioned above may support the contention that caspase-8 activation through the extrinsic pathway is involved in EEER-induced apoptosis.

In conclusion, we found that EEER had a potent inhibitory activity toward the 5α-reductase enzyme and suppressed the growth of BPH-1 cells via the induction of caspase-dependent apoptosis. Given that EEER increased the activity of caspase-3 and caspase-8, but not caspase-9, we suggest that EEER causes apoptosis in BPH-1 cells through the extrinsic pathway. Taken together, these findings provide evidence of the potential of *E. rutaecarpa* Bentham as a phytotherapeutic agent against BPH.

## Figures and Tables

**Table 1:** Inhibitory effects of ethanol extract of *E. rutaecarpa* (EEER) and finasteride on rat prostate testosterone 5α-reductase.

Treatment	Concentrations		
	Inhibition (%) at a Concentration of Extract		
Finasteride	0.372 ng/mL	3.72 ng/mL	37.2 ng/mL
	20.1 ± 14.6	68.6 ± 4.8	89.3 ± 15.4
EEER	25 μg/mL	100 μg/mL	250 μg/mL
	80.2 ± 11.4	94.3 ± 10.7	104.1 ± 4.8

The values are the means of the results from two independent experiments in duplicate.
